# Emerging Role of Circular RNAs in Cancer

**DOI:** 10.3389/fonc.2020.00663

**Published:** 2020-05-20

**Authors:** Jing Liu, Xin Zhang, Meinan Yan, Hui Li

**Affiliations:** ^1^Department of Gastrointestinal Cancer Biology, Tianjin Medical University Cancer Institute and Hospital, Tianjin, China; ^2^Key Laboratory of Cancer Immunology and Biotherapy, Tianjin, China; ^3^National Clinical Research Center for Cancer, Tianjin, China

**Keywords:** circular RNAs, molecular mechanism, cancer, clinical application, biomarker

## Abstract

Circular RNAs (circRNAs), which are generated mainly from back-splicing of exons in precursor mRNAs (pre-mRNAs), are a novel class of endogenous covalently closed RNA molecules. Their functions as microRNA sponges, protein scaffolds, and modulators of transcription and splicing, as well as occasional templates for polypeptide production, are beginning to be recognized, though the investigation of circRNAs is in its infancy. circRNAs play critical roles in diverse cellular processes. Aberrant expression of circRNAs in malignancies sustains cellular growth and proliferation, promotes cellular invasiveness, and circumvents cellular senescence and death, suggesting their potential for exploitation as clinical biomarkers and therapeutic targets. In this review, we highlight recent progress in research on circRNAs in cancer, emphasizing the molecular mechanisms and potential clinical value of circRNAs.

## Introduction

Circular RNAs (circRNAs), a novel class of endogenous covalently closed RNA molecules, have attracted great attention in the past few years. They are generally derived from back-splicing of precursor mRNA (pre-mRNA), during which the 3′ splice donor sequence is joined to the downstream 5′ splice acceptor sequence (1, 2). In 1976, Sanger et al. (3) first identified the single-stranded viroid RNA in circular formats with high thermal stability *via* electron microscopy. Subsequently, this kind of circRNA transcript was found in hepatitis delta virus (4), yeast (5), archaea (6), fruit flies (7), and mammals (8). In 1991, Nigro et al. (9) discovered the presence of circRNA transcripts derived from the Deleted in Colon Cancer (DCC) gene in humans for the first time; subsequently, other genes, including ETS-1 gene (10), the human cytochrome P450 gene (11), the human dystrophin gene (12), and the antisense noncoding RNA in the INK4 locus (13), were identified to produce circular transcripts. However, these circRNAs were long considered as aberrant splicing by-products with low abundance and limited biological function (14).

With the development of high-throughput RNA sequencing (RNA-seq) and bioinformatics tools, numerous circRNAs have been identified. Salzman et al. (15) explored the circRNA map in pediatric acute lymphoblastic leukemia samples and revealed that large portions of spliced gene transcripts are circRNAs. Jeck et al. (16) identified >25,000 circRNAs that are not degraded by exonucleases in human fibroblasts. Circular splicing of RNA is accepted to be a general feature of gene expression, but whether these circRNAs are functional is a primary concern of researchers.

Studies to date have reported that circRNAs are dysregulated in the pathophysiologic processes of several diseases, including cardiovascular disease (17), neurodegenerative disease such as Alzheimer's disease (18), metabolic disorder (19), diseases caused by viral infection (20), and cancer, which is the focus of the following sections (21). Abnormal expressed circRNAs can modulate gene transcription *via* indirect interactions with other transcription factors, such as microRNAs (miRNAs) and RNA binding proteins (RBPs) (22, 23). Sporadic studies have also pointed out that some circRNAs containing translation initiation elements can be translated into functional proteins and peptides (24, 25). Moreover, the competitive splicing mechanism of circRNAs also affects the expression of their parent genes, thus producing biological effects (26). In addition to functioning as regulators of gene expression, circRNAs are also novel promising biomarkers for disease diagnosis and prognosis assessment due to their stable closed circular structure and tissue- and developmental stage-specific expression patterns (27, 28).

However, the understanding of circRNAs is in its infancy, and knowledge of the biological characteristics of these molecules requires further supplementation. More work is needed to explore the emerging roles of circRNAs in cancer. In this review, we introduce the biogenetic model, expression profile, and functional mechanism of circRNAs and summarize recent progress in circRNA research and their application in cancer.

## Mechanisms for circRNA Generation

circRNAs are divided into three main types according to their sources, namely, exonic circular RNAs (ecircRNAs), circular intronic RNAs (ciRNAs), and exon–intron circular RNAs (elciRNAs), among which ecircRNAs are the most common (29). Several circularization mechanisms produce circRNAs. These elements shorten the spatial distance between the two ends of the loop sequence and provide possibilities for back-splicing.

Intron pairing-driven circularization is mediated by 30–40 nucleotide (nt) reverse complementary sequences in the flanking regions of circularized exons; these sequences form double-stranded RNA structures and therefore promote circRNA production as cis-acting elements (2, 16). Alu is the most common reverse complementary element in mammals. In addition, some specific RBPs can bind to both sides of flanking intron sequences and bring splice donors and splice acceptors sufficiently close through protein–protein interactions (30), such as Quaking (QKI) (31), NF90/NF110 (32), and FUS (33). In contrast, other RBPs may inhibit circRNA production. For example, adenosine deaminase acting on RNA 1 (ADAR1), a common Alu editing element, was identified to deaminate adenosine nucleosides to inosine (A-to-I editing) in regions that complementary and proximal to the splice sites of circularized exons and destabilize intron pairing interactions, thereby antagonizing circRNA biogenesis (34). Lariat-driven circularization occurs in the process of exon skipping. This process produces a lariat intermediate containing introns and exons, which then undergo back-splicing to form circRNAs. The production of ciRNAs is a special situation in the lariat-driven model (35). CircRNA biogenesis *in vivo* is indeed very complicated. The expression level of a circRNA may be influenced by various circularization mechanisms. In addition, alternative splicing is a key component of circRNA production and gene expression regulation (36, 37).

## circRNA Expression and Characteristics

circRNAs have been identified to be expressed widely in most organisms. The size of circRNAs ranges from <100 to several thousand nucleotides (30), and the common size reported in human cells is a few hundred nucleotides comprising 2–3 exons. The expression of most circRNAs is generally lower than that of their linear transcripts (38), but a few circular transcripts exhibit an expression level slightly or much higher than those of the their linear transcripts, such as the products of the CDR1 and Sry genes (8, 15, 16, 22). In addition, due to their unique closed circular structure, circRNAs are protected from degradation by exonucleases and are more stable than linear RNAs (16). Multiple studies have reported that circRNAs have longer half-lives than linear transcripts both *in vitro* and *in vivo* (39, 40). Moreover, circRNA expression is generally tissue- and developmental stage-specific. Several studies have shown that many circRNAs are upregulated in the nervous system (41–44), a characteristic that may be related to their posttranscriptional accumulation in neurons (45–47). Therefore, scientists hypothesized that the intracellular level of circRNAs was negatively correlated with the cell proliferation index (48). This hypothesis may explain why circRNA expression in tumor cells is generally lower than that in normal cells. Moreover, circRNAs may have higher sequence conservation than other types of RNA in mammals (41). Taken together, the high conservation, stability, and specificity of circRNAs imply that circRNAs may have multiple biological functions and clinical applications and are unlikely to be simply splicing by-products.

## Techniques for Studying circRNAs

Although circRNAs were discovered decades ago, they could not be detected by early routine poly(A)-enriched RNA sequencing technology. Panda et al. (49) proposed a novel method, RNase R treatment followed by polyadenylation and poly (A)+ RNA depletion (RPAD), to enrich highly pure circRNAs. In this method, total RNAs are first treated with RNase R to deplete linear RNAs. The remaining RNAs with 3'-OH ends are polyadenylated and removed by poly (A)+ RNA depletion *via* oligo (dT) beads. The RPAD method eliminated the interference of linear RNAs and significantly improved the reliability of the data. However, RPAD is unsuitable for joint analysis of circRNAs with other molecules such as miRNAs and mRNAs. Researchers should adopt different methods to optimize RNA sequencing libraries for the specific experimental purpose.

To date, a variety of algorithms have been developed to identify circRNAs. CircRNAs are generated primarily from pre-mRNA back-splicing, not canonical splicing; thus, the mapping algorithms used in early transcriptome analysis cannot directly match the fragments to the genome. Therefore, sequencing reads that span back-splicing sites require further genomic alignment and correction (50, 51). Various bioinformatics algorithms have been developed for circRNA annotation and quantification (51, 52). The use of two or more tools simultaneously is recommended to meet specific research demands for circRNA identification (52). In addition, multiple databases have been developed for circRNA analysis (Table 1). Through these databases, researchers can search basic information about circRNAs, predict interactions of circRNAs with target molecules and their translation potential, and evaluate their relationships with diseases.

**Table 1:** Databases about circRNAs.

**Database**	**Website**	**Function**	**References**
Circbase	http://www.circbase.org/	Circbase collects and integrates published circRNA data and identifies circRNAs in sequencing data by find_circ software	(53)
CIRCpedia v2	http://www.picb.ac.cn/rnomics/circpedia	CIRCpedia contains transcriptome data from approximately 180 samples across six species and systematically annotates alternative back-splicing and alternative splicing events of circRNAs	(36)
DeepBase v2.0	http://rna.sysu.edu.cn/deepBase/	DeepBase v2.0 collects a total of 107,913 circRNAs, of which 92.5% were found in human	(54)
CircRNADb	http://202.195.183.4:8000/circrnadb/circRNADb.php	CircRNADb provides not only basic information about the chromosomal position and sequences of circRNAs but also the information about their protein-coding potential	(55)
TSCD	http://gb.whu.edu.cn/TSCD	TSCD collects tissue-specific circular RNAs in humans and mice	(56)
CircInteractome	http://circinteractome.nia.nih.gov/	CircInteractome provides information about microRNA and RBP binding sites on circRNAs and can design specific primers and siRNA for circRNA experiment verification	(57)
CSCD	http://gb.whu.edu.cn/CSCD/	CSCD collects circRNA data from 87 cancer cell lines and 141 normal cells, describing cancer-specific circRNA	(58)
Circ2Traits	http://gyanxet-beta.com/circdb/	Circ2Traits integrates 1,954 circular RNAs related to human diseases. The included circRNA contains disease-related SNPs or can interact with disease-related miRNAs	(59)
MiOncoCirc	https://nguyenjoshvo.github.io/	MiOncoCirc collects the data of circRNA expression in different cancer clinical samples	(60)
ExoRBase	http://www.exorbase.org/	ExoRBase collects 58330 circRNAs from 92 serum exosomal RNA sequencing samples	(61)

*circRNA, circular RNA; RBP, RNA binding protein*.

## Functions of circRNAs

Numerous reports have confirmed that circRNAs can regulate gene expression directly or, more commonly, through binding with miRNAs, RBPs, and other gene expression regulators, thereby regulating various biological processes.

### miRNA Sponging

miRNAs are a large class of small (~22 nt) noncoding single-stranded RNAs that can bind to their target mRNAs to inhibit their translation or promote their degradation. Recent studies have reported that many circRNAs can act as competitive endogenous RNAs (ceRNAs), binding with miRNAs through miRNA response elements (MREs) to downregulate the function of the target miRNA. The most representative example is CDR1as (antisense to the cerebellar degeneration-related protein 1 transcript), also known as ciRS-7 (22, 62). CDR1as contains 73 binding sites for miR-7, and the interaction of these two RNAs can inhibit the function of miR-7. In addition, their interaction provides a novel mechanism for miR-7 transport. Similarly, circSry, derived from the mouse sex-determining gene Sry, contains 16 binding sites for miR-138 and can act as a miRNA sponge (29). Notably, most ecircRNAs are localized mainly in the cytoplasm, indicating their availability to bind with miRNAs and regulate their function (39). However, most circRNAs do not contain multiple miRNA binding sites (29). Although this mechanism is the most widely studied mechanism of circRNAs, its importance remains to be confirmed. The abundance of most circRNAs is generally low, which also limits the universality of the miRNA sponge hypothesis.

### Protein Scaffolding

CircRNAs contain many RBP binding sites in addition to MREs. For example, human antigen R (HuR) has been reported to bind with numerous circRNAs in human cervical carcinoma HeLa cells (63). CircPABPN1, derived from the PABPN1 gene, can compete with PABPN1 mRNA for binding to HuR, thereby inhibiting the translation of PABPN1. In addition, elciRNAs and ciRNAs, predominantly localized in the nucleus, were shown to be able to interact with small nuclear ribonucleoprotein U1 (snRNP U1) and enhance RNA polymerase II (Pol II) transcriptional activity on their parental gene as cis regulators (35, 64). Additionally, the binding of circRNAs to some functional proteins may affect multiple signaling pathways leading to homeostasis changes. Du et al. (65) found that the circRNA circ-Foxo3 interacted with the anti-senescence protein ID-1, the transcription factor E2F1, and the anti-stress proteins FAK and HIF1α and retained them in the cytoplasm to hinder their corresponding functions.

### Translational Templates

Although circRNAs have historically been considered noncoding RNAs, several recent studies have indicated that some circRNAs contain translation initiation sites and have translational potential (39). For example, some circRNAs with internal ribosome entry sites (IRESs) can be translated into proteins *in vitro* and *in vivo* (24). FBXW7-185aa is a novel protein encoded by circ-FBXW7 and contains an IRES (66). N6-methyladenosine(m6A), the most common RNA base modification, is abundant in circRNA sequences and can facilitate translation initiation by recruiting eIF4G2 and YTHDF3 (67). Another study in 2017 found that circ-ZNF609 contained an open reading frame and could be translated in human skeletal muscle. To date, only a few circRNAs have been reported to be involved in the translation process. Although some bioinformatics tools have been developed to predict the translation potential of circRNAs, their accuracy needs to be further improved and experimentally verified. In addition, whether these proteins or peptides formed from circRNAs have important functions needs exploration.

### Other Functions

Moreover, the biosynthesis of circRNAs can compete with the splicing of their linear transcripts, thus affecting their expression and corresponding functions (68). Because of their stability, circRNAs can also accumulate over time and may thus act as memory molecules for the transcriptional history of a cell (69). The presence of circRNAs in vesicles also suggests their function as signaling molecules (70).

## The Functions of circRNA and Cancer

CircRNAs are abnormally expressed in many cancers, such as lung cancer, breast cancer, digestive system cancers, ovarian cancer, and glioblastoma. Hang et al. (71) identified 185 differentially expressed circRNAs between non-small-cell lung cancer (NSCLC) tissues and adjacent normal tissues through RNA sequencing. In addition, Zeng et al. (72) detected 192 upregulated and 239 downregulated circRNAs in colorectal cancer (CRC) tissues from patients with or without pulmonary metastasis. Whether this differential expression is related to cancer development has been a focus of circRNA research.

### Cell Proliferation

*Via* high-throughput sequencing, Xie et al. (73) screened a circRNA related to bladder cancer (BC), BCRC-3. BCRC-3 is poorly expressed in BC tissues and cell lines, and it can bind to miR-182-5p to act as a ceRNA, thereby upregulating the expression of p27 and inhibiting BC cell proliferation. Liang et al. (74) found that circβ-catenin was upregulated in liver cancer tissues compared to adjacent tissues. Silencing circβ-catenin significantly inhibited malignant phenotypes. Mechanistically, circβ-catenin can be translated into a 370-amino acid (aa) β-catenin isoform. This β-catenin−370aa construct competed with GSK3β to inhibit its degradation of β-catenin, thereby activating the Wnt/β-catenin pathway in liver cancer to promote tumor growth. Moreover, circACC1 has been reported to bind the regulatory β and γ subunits of AMP-activated protein kinase (AMPK), resulting in a ternary complex, which in turn activates AMPK enzyme activity and then promotes fatty acid b-oxidation and glycolysis (75). Overexpression of circACC1 can promote tumorigenesis. A positive correlation between AMPK activation and elevated circACC1 expression was identified in CRC tissues. Zhang et al. (76) revealed that circNRIP1 can sponge miR-149-5p and thus affect the AKT1/mammalian target of rapamycin (mTOR) axis, acting as a tumor promoter in gastric cancer (GC). This study also suggested that circNRIP1 can assemble into exosomes and participate in exosomal communication among GC cells (76).

### Invasion and Metastasis

Recently, Hu et al. (77) identified a circRNA, circASAP1, that is associated with pulmonary metastasis after curative resection in hepatocellular carcinoma (HCC) patients. Studies have shown that circASAP1 acts as a sponge of miR-326 and miR-532-5p, which have mitogen-activated protein kinase (MAPK)1 and colony stimulating factor (CSF-1) in common as target genes, thereby promoting HCC cell invasion and macrophage infiltration. In addition, Chen et al. (78) discovered a circRNA, FECR1, derived from Friend leukemia virus integration 1 (FLI1). Overexpression of FECR1 can increase the invasiveness of breast cancer cells. Mechanism studies have shown that FECR1 can recruit TET1 and act in trans to downregulate DNMT1, causing DNA demethylation of FLI1 and promoting cell metastasis. FECR1 may be a potential therapeutic target for metastatic breast cancer. m6A-modified circNSUN2 can form a ternary complex with insulin-like growth factor 2 mRNA binding protein 2 (IGF2BP2) and high-mobility group A2 (HMGA2) to enhance HMGA2 mRNA stability and subsequently promote liver metastasis in CRC (79). CircFoxo3 expression is low in high-grade prostate cancer, and overexpression of circFoxo3 in DU145 cells can inhibit the epithelial–mesenchymal transition (EMT) and reduce cell viability by enhancing Foxo3 expression (26). CircMTO1 (hsa_circRNA_104135) is significantly downregulated in HCC and can affect p21 expression by targeting miR-9 and, in turn, promote cell proliferation and invasion (80).

### Cell Cycle

Circ-Foxo3 has been shown to bind with CDK2 and p21, leading to formation of the circ-Foxo3–p21–CDK2 ternary complex (81). CDK2 generally interacts with cyclin A/E to promote cell cycle progression, while p21 inhibits these effects. Formation of this ternary complex enhanced the inhibitory effect of p21 on CDK2, which prevented cell cycle transition from G1 to S phase and thus inhibited cell proliferation. Cheng et al. (82) found significant upregulation of circTP63 in lung squamous cell carcinoma (LUSC) tissues. CircTP63 competitively binds to miR-873-3p, thereby abolishing the inhibitory effect of miR-873-3p on its target gene Forkhead Box M1 (FOXM1). Elevated FOXM1 expression further enhances the expression of centromere protein (CENP)A and CENPB, ultimately promoting cell cycle progression and cell proliferation.

### Cell Death

Circ-Foxo3 was reported to be downregulated in tumor tissues and cells (83). On the one hand, transfection of circ-Foxo3 can induce apoptosis as a stress stimulus. On the other hand, highly expressed circ-Foxo3 interacts with p53 and murine double minute 2 (MDM2) and inhibits ubiquitination-mediated degradation of Foxo3 by MDM2. Overexpression of Foxo3 promotes apoptosis *via* its target Puma. Du et al. (84) detected abnormally increased circ-Dnmt1 expression in breast cancer tissues and cells and found that circ-Dnmt1 interacted with p53 and AUF1, leading to their nuclear translocation, thus mediating cell autophagy. Transfection of circ-Dnmt1 into breast cancer cells can induce autophagy to maintain cell homeostasis and ultimately promote cell proliferation and tumorigenesis. Furthermore, Chen et al. (85) found that circHIPK3 expression was upregulated in STK11 mutant lung cancer cell lines. Silencing circHIPK3 can induce autophagy through the miR124-3p–STAT3–PRKAA/AMPKa axis. Thus, circHIPK3 is a key upstream regulator of autophagy in lung cancer.

### Others

CircRNAs can also regulate the drug sensitivity of tumors. By high-throughput sequencing of circRNAs of five pairs of cisplatin-sensitive and cisplatin-resistant ovarian cancer tissues, Zhao et al. (86) found that CDR1as was significantly downregulated in cisplatin-resistant tissues. Mechanism studies confirmed that CDR1as can regulate the sensitivity of ovarian cancer to cisplatin through the miR-1270/SCAI signaling pathway and promote ovarian cancer development. In addition, circRNAs derived from fusion genes have been shown to play a special role in cancers. Recent studies have demonstrated that chromosomal translocations in cancer could not only encode oncogenic fusion proteins but also generate circRNAs. This kind of fusion circRNA (f-circRNA) is unique to cancer and may play an important role in tumorigenesis. F-circRNAs were first identified in a study on leukemia, in which F-circPR and F-circM9 (derived from PML-RARα and MLL-AF9 fusion genes, respectively) were found to promote cellular transformation, cell viability, and drug resistance (87). In addition, F-circEA-2a, derived from the EML4-ALK fusion gene in NSCLC, is located mainly in the cytoplasm and can promote cell migration and invasion (88). The tumor-suppressive effect of circFAT1 (e2) in GC can simultaneously arise through the cytoplasmic circFAT1 (e2)/miR-548g/RUNX1 axis and the nuclear circFAT1 (e2)/YBN1 regulatory network (89). Thus, the mechanism of circRNAs *in vivo* is very complicated, and our current knowledge of this process is only nascent. Additional studies are listed in Table 2.

**Table 2:** A list of circRNAs related to cancer.

**Cancer type**	**CircRNA**	**Mechanism**	**Target**	**Expression in cancer**	**Function**	**References**
Lung cancer	hsa_circ_0007059	MiRNA sponge	miR-378/Wnt/β-catenin miR-378/ ERK1/2	Down	Inhibit cell proliferation and EMT, promote apoptosis	(90)
	hsa_circ_103809	MiRNA sponge	miR-4302/ZNF121/MYC	Up	Promote cell proliferation and invasion	(91)
	circRNA 100146	MiRNA sponge	miR-361-3p, miR-615-5p/SF3B3	Up	Inhibit cell proliferation and invasion, promote apoptosis	(92)
	circPTK2	MiRNA sponge	miR-429, miR-200b-3p/ TIF1γ/TGF-β	Down	Inhibit EMT and metastasis	(93)
	circHIPK3	MiRNA sponge	miR124-3p/STAT3/PRKAA and AMPKa	Up	Promote cell proliferation, migration and invasion, inhibit macroautophagy and autophagy	(85)
	cESRP1	MiRNA sponge	miR-93-5p/CDKN1A /TGF-β	Down	Increase chemotherapy sensitivity	(94)
	cMras	MiRNA sponge	miR-567/PTPRG	Down	Inhibit LUAD growth and metastasis	(95)
Colorectal cancer	circNSUN2	Protein scaffolds	IGF2BP2/ HMGA2	Up	Promote liver metastasis and cells invasion	(79)
	circACC1	Protein scaffolds	AMPK	Up	Promote cell proliferation	(75)
	circCCDC66	MiRNA sponge	miR-33b, miR-93	Up	Promote cell proliferation, migration, and metastasis	(96)
	circHIPK3	MiRNA sponge	miR-7/ FAK, IGF1R, EGFR, YY1	Up	Promote cell proliferation, migration and invasion, inhibit cell apoptosis	(97)
	circPPP1R12A	Translation template	circPPP1R12A-73aa/Hippo-YAP	Up	Promote cell proliferation, migration and invasion	(25)
	circLgr4	Translation template	circLgr4-peptide /Wnt/β-catenin	Up	Promote colorectal cancer stem cells (CSCs) self-renewal, colorectal tumorigenesis and invasion	(98)
	hsa_circ_0053277	MiRNA sponge	miR-2467-3p/MMP14	Up	Promote cell proliferation, migration, and EMT	(99)
	hsa_circ_0001178	MiRNA sponge	miR-382, miR-587, miR-616/ZEB1	Up	Promote cell migration and invasion	(100)
Gastric cancer	circNRIP1	MiRNA sponge	miR-149-5p/AKT1-mTOR	Up	Promote cell proliferation, migration, invasion	(76)
	circPSMC3	MiRNA sponge	miR-296-5p/PTEN	Down	Inhibit the tumorigenesis *in vivo* and *in vitro*	(101)
	circ-DONSON	Protein scaffolds	NURF/SOX4	Up	Promote cell proliferation, migration and invasion, inhibit apoptosis	(102)
	circYAP1	MiRNA sponge	miR-367-5p/p27 Kip1	Down	Inhibit cell growth and invasion *in vitro* and *in vivo*	(103)
	circLARP4	MiRNA sponge	miR-424/LATS1	Down	Inhibit cell proliferation and invasion	(104)
	circFAT1(e2)	MiRNA sponge	miR-548g/RUNX1 YBX1	Down	Promote cell proliferation, migration, and invasion	(89)
	circDLST	MiRNA sponge	miR-502-5p/NRAS/MEK1/ERK1/2	Up	Promote the tumorigenesis and metastasis	(105)
	circ-HuR	Protein scaffolds	CNBP/HuR	Down	Inhibit cell growth, invasion, and metastasis	(106)
	circOSBPL10	MiRNA sponge	miR-136-5p/WNT2	Up	Promote cell growth, migration, and invasion	(107)
Breast cancer	circKIF4A	MiRNA sponge	circKIF4A-miR-375-KIF4A	Up	Promote cell proliferation and migration	(108)
	FECR1	Protein scaffolds	FLI1/TET1	Up	Enhance invasiveness of breast cancer cells	(78)
	circ-Dnmt1	Protein scaffolds	p53/AUF1	Up	Promote the proliferative and survival capacities of breast cancer cells by stimulating cellular autophagy	(84)
	circANKS1B	MiRNA sponge	miR-148a-3p, miR-152-3p/USF1/TGF-β1/Smad	Up	Promote breast cancer invasion and metastasis	(109)
	circEPSTI1	MiRNA sponge	miR-4753, 6809/BCL11A	Up	Promote cell proliferation, inhibit apoptosis	(94)
	circIRAK3	MiRNA sponge	miR-3607/FOXC1	Up	Promote cell migration, invasion and metastasis	(110)
	circAGFG1	MiRNA sponge	miR-195-5p/ CCNE1	Up	Promote triple-negative breast cancer (TNBC) cell proliferation, mobility and invasion as well as tumorigenesis and metastasis *in vivo*	(111)
	circFBXW7	Translation template	FBXW7-185aa/c-Myc	Down	Inhibit proliferation and migration abilities of TNBC cells	(112)
HCC	cSMARCA5	MiRNA sponge	miR-17-3p, miR-181b-5p/TIMP3	Down	Inhibit cell proliferation and migration of HCC cells	(113)
	circMTO1	MiRNA sponge	miR-9/p21	Down	Inhibit cell proliferation and invasion	(80)
	circ-CDYL	MiRNA sponge	miR-892a/HDGF, miR-328-3p/HIF1AN	Up	Promote tumor growth	(114)
	circβ-catenin	Translation template	β-catenin/Wnt	Up	Promote malignant phenotypes *in vitro* and *in vivo*	(74)
	circSLC3A2	MiRNA sponge	miR-490-3p/PPM1F	Up	Promote HCC cell proliferation and invasion	(115)
	circHIPK3	MiRNA sponge	miR-124/AQP3	Up	Promote cell proliferation and migration	(116)
	circRHOT1	Protein scaffolds	TIP60/NR2F6	Up	Promote proliferation and metastasis	(116)
	circTRIM33-12	MiRNA sponge	miR-191/TET1	Down	Inhibit proliferation, migration, invasion and immune evasion abilities of HCC cells	(117)
Ovarian cancer	circPLEKHM3	MiRNA sponge	miR-9/BRCA1/DNAJB6 /KLF4/AKT1	Down	Inhibit cell growth, migration and EMT	(118)
	hsa_circ_0061140	MiRNA sponge	miR-370/FOXM1	Up	Promote cell proliferation and migration	(119)
	hsa_circ_0051240	MiRNA sponge	miR-637/KLK4	Up	Promote cell proliferation, migration and invasion	(120)
	circWHSC1	MiRNA sponge	miR-145/ MUC1 miR-1182/hTERT	Up	Promote cell proliferation, migration and invasion, inhibit cell apoptosis	(121)
Prostate cancer	circFoxo3	Modulators of transcription	Foxo3	Down	Inhibit prostate cancer viability and EMT	(26)
	circZNF609	MiRNA sponge	miR-186-5p/YAP1/AMPK	Up	Promote growth, migration and invasion	(122)
	circ0005276	Modulators of transcription	FUS/XIAP	Up	Promote cell proliferation, migration and EMT	(123)
Pancreatic cancer	circZMYM2	MiRNA sponge	miR-335-5p/JMJD2C	Up	Promote cell proliferation and invasion, inhibit cell apoptosis	(124)
	hsa_circ_0000977	MiRNA sponge	miR-874-3p/PLK1	Up	Promote pancreatic cancer growth	(125)
	circ-ASH2L	MiRNA sponge	miR-34a/Notch 1	Up	Promote tumor invasion, proliferation and angiogenesis	(126)
Glioblastoma	circNT5E	MiRNA sponge	miR-422a	Up	Promote cell proliferation, migration and invasion	(127)
	circMMP9	MiRNA sponge	miR-124/CDK4/AURKA	Up	Promote cell proliferation, migration and invasion	(128)
Bladder cancer	BCRC-3		miR-182-5p/p27	Down	Inhibit cell proliferation	(73)
Esophageal squamous cell carcinoma	ciRS-7	MiRNA sponge	miR-7/HOXB13/NF-κB/p65	Up	Inhibit cell proliferation, migration and invasion	(129)
Gallbladder cancer	circERBB2	Protein scaffolds	PA2G4/TIFIA	Up	Modulate ribosomal DNA transcription and cell proliferation	(130)

*circRNA, circular RNA; EMT, epithelial–mesenchymal transition; HCC, hepatocellular carcinoma; LUAD, lung adenocarcinoma*.

## circRNAs as Biomarkers in Cancer

CircRNAs have a covalently closed circular structure, which increases their resistance to exonuclease digestion and their accumulation in body fluids and tissues (131, 132). In addition, circRNAs are often expressed in a tissue- and developmental stage-specific manner. The properties of circRNAs have inspired numerous studies on their application as promising biomarkers in cancer.

### Diagnostic Biomarkers

#### circRNAs in Tissues

Wei et al. (114) found that circ-CDYL and its target genes (HDGF and HIF1AN) were highly expressed in Barcelona Clinic Liver Cancer (BCLC) stages 0 and A of HCC. Their sensitivity and specificity as combined biomarkers were higher than those of alpha-fetoprotein (AFP) in the early stage of HCC. These results indicated that a panel combining circ-CDYL with HDGF and HIF1AN could be used as a monitoring indicator for early HCC or high-risk populations. In addition, Zhong et al. (133) detected highly expressed CDR1as in nasopharyngeal carcinoma (NPC) biopsy samples. However, unfortunately, current methods for detecting circRNAs in tissue are complex and invasive, which greatly limit their roles in the early diagnosis and screening of cancer.

#### circRNAs in Peripheral Blood

Numerous studies have confirmed that circRNAs can be stably enriched in peripheral blood, urine, and saliva. Therefore, circRNAs could become suitable biomarkers for liquid biopsy. Recently, a study on the use of plasma circRNAs in the diagnosis of hepatitis B virus (HBV)-associated HCC was published (134). This study included 1,195 plasma samples, which were divided into a training set and two validation sets. The researchers found that the plasma expression levels of hsa_circ_0000976, hsa_circ_0007750, and hsa_circ_0139897 in HCC patients were significantly higher than those in healthy controls and patients with chronic hepatitis B or HBV-related liver cirrhosis. They also designed an HCC prediction model named CircPanel through binary logistic regression analysis. CircPanel showed higher sensitivity and specificity (both higher than 80%) than AFP level for distinguishing HCC patients from controls. Notably, CircPanel can also diagnose AFP-negative HCC and AFP-negative small HCC (solitary, diameter ≤3 cm) with high diagnostic accuracy. In another study, Lin et al. (135) found that the plasma levels of circ-CCDC66, circ-ABCC1, and circ-STIL were significantly decreased in CRC patients compared with controls. The combination of these three circRNAs had a sensitivity and specificity of 64.4% and 85.2%, respectively, for diagnosing CRC. Notably, f-circRNAs derived from fusion genes are generally cancer-specific and thus have high specificity in diagnosing cancer, which is a unique advantage for tumor biomarkers. Tan et al. (136) reported a fusion circRNA named F-circEA derived from an EML4-ALK fusion gene that was positively expressed in five of six NSCLC patients with EML4-ALK translocation. F-circEA also exists specifically in the plasma of EMLA4-ALK-positive NSCLC patients.

#### circRNAs in Exosomes

Exosomal circRNAs have received gradually increasing attention in recent years. Li et al. (137) identified more than 1,000 circRNAs in human serum exosomes for the first time and suggested that these circRNAs could distinguish between colon cancer patients and healthy controls. Pan et al. (138) used qRT-PCR to detect hsa-circ-0004771 in circulating exosomes from 170 patients and 45 healthy controls and confirmed that hsa-circ-0004771 could clearly distinguish between patients with stage I/II CRC and those with benign intestinal diseases. Li et al. (139) detected high expression of circ-PDE8A in liver-metastatic pancreatic ductal adenocarcinoma (PDAC) tissues, and this characteristic was confirmed to be closely related to lymphatic infiltration, T status, and TNM stage. Circ-PDE8A is an independent risk factor for the survival of PDAC patients. Further research confirmed the presence of circ-PDE8A-rich exosomes secreted from tumor cells in the plasma of PDAC patients. Similarly, circ-PDE8A in plasma exosomes may also be a diagnostic and prognostic marker for PDAC.

#### circRNAs in Other Body Fluids

The use of circRNAs in other body fluid samples has also been studied. For example, gastric juice examination is a highly organ-specific test for the diagnosis of gastric diseases. Shao et al. (140) explored the feasibility of hsa_circ_0014717 in gastric juice as a biomarker for screening patients with GC. The expression of hsa_circ_0014717 in gastric juice from 38 healthy people, 30 patients with gastric ulcers, 15 patients with chronic atrophic gastritis, and 39 patients with GC was measured by qRT-PCR. Hsa_circ_0014717 was significantly downregulated in patients with chronic atrophic gastritis compared with healthy controls, suggesting the potential utility of hsa_circ_0014717 in gastric juice as a biomarker for screening high-risk populations for GC. In addition, saliva is a body fluid used for disease research due to its convenient and noninvasive sampling method. Bahn et al. (141) detected and verified the presence of circRNAs in cell-free saliva, which suggested a new direction for the application of circRNAs as biomarkers. Moreover, Chen et al. (142) detected highly expressed circPRMT5 in exosomes isolated from urine samples from patients with urothelial carcinoma of the bladder (UCB). Additional studies are listed in Table 3.

**Table 3:** CircRNAs as diagnostic biomarkers in cancer.

**Cancer type**	**CircRNA**	**Source**	**Cohort size**	**Expression in cancer**	**AUC**	**Sensitivity %**	**Specificity %**	**Relationship with other biomarkers**	**References**
HCC	circZKSCAN1	Tissue	102	Down	0.834	82.2	72.4	–	(143)
	circ_104075	Tissue	89	Down	0.973	96.0	98.3	Perform better than DANCR, HULC, miR-223, miR-21, UCA1, AFP, DCP, and AFP-L3-	(144)
	circSMARCA5	Plasma	489	Down	0.938	–	–	When combined with AFP, the AUC increased to 0.903 and 0.858 in detecting HCC from hepatitis and cirrhosis, respectively	(145)
	hsa_circ_0000976, hsa_circ_0007750 and hsa_circ_0139897	Plasma	1195	Up	0.863 0.843 0.864	–	–	Perform better than AFP in diagnosing HCC and SmallHCC	(134)
	Circ-CDYL	Tissue	149	Up	0.73	75.36	66.67	Perform better than AFP in diagnosing early stage of HCC	(114)
Gastric cancer	hsa_circ_0000745	Plasma	120	Down	0.683	85.5	45.0	When combined with CEA, the AUC increased to 0.775, while the sensitivity and specificity changed to 0.800 and 0.633	(146)
	hsa_circ_0000520	Tissue	56	Down	0.6129	53.57	85.71	–	(147)
	hsa_circ_0000520	Plasma	62	Down	0.8967	82.35	84.44	Associated with CEA	(147)
	hsa_circ_0014717	Tissue	96	Down	0.696	59.38	81.25	Associated with CEA and CA19-9	(140)
	hsa_circ_0000096	Tissue	101	Down	0.82	–	–	When combined with hsa_circ_002509, the AUC increased to 0.91, while the sensitivity and specificity changed to 0.800 and 0.633	(148)
Lung cancer	hsa_circ_0013958	Tissue	49	Up	0.815	75.5	79.6	–	(149)
	hsa_circ_0075930	Tissue	92	Up	0.756	76.2	72.1	–	(150)
	circRNA_102231	Tissue	57	Up	0.897	81.2	88.7	–	(151)
	circFARSA	Plasma	100	Up	0.71	–	–	–	(71)
Colorectal cancer	circ-CCDC66, circ-ABCC1and circ-STIL	Plasma	106	Down	0.78	64.4	85.2	When combined with CEA and CA19-9, the AUC increased to 0.855 in detecting CRC	(135)
	circVAPA	Plasma	103	Up	0.724	–	–	–	(152)
	circITGA7	Tissue	69	Down	0.8791	92.75	66.67	–	(153)
	hsa-circ-0004771	Circulating exosome	215	Up	0.88	80.91	82.86	–	(138)
Pancreatic cancer	circ-LDLRAD3	Plasma	62	Up	0.67	57.38	70.49	When combined with CA19-9, the AUC was increased to 0.87 and the sensitivity and specificity were 0.8033 and 0.9355, respectively	(154)
Osteosarcoma	circPVT1	Serum	90	Up	0.871	–	–	Perform better than ALP and LDH	(155)

*AFP, alpha-fetoprotein; circRNA, circular RNA; HCC, hepatocellular carcinoma*.

### Prognostic Biomarkers

#### circRNAs Predict Patient Survival

CircRNAs have wide application prospects in monitoring the treatment efficacy and assessing the prognosis of cancer. By using RNA-seq to identify circRNAs related to HCC progression, Qiu et al. (156) determined that circADAMTS13 was significantly downregulated in HCC tissues compared with matched nontumor tissues. Moreover, its expression was positively associated with recurrence-free survival (RFS). Receiver operating characteristic (ROC) curve analysis was used to evaluate the diagnostic value of circADAMTS13 in HCC patients, and the area under ROC curve (AUC) was 0.987. He et al. (157) determined that circGFRA1 was significantly upregulated in triple-negative breast cancer via circRNA microarray analysis. In addition, Kaplan–Meier survival analysis showed that upregulation of circGFRA1 was associated with poorer clinical survival. CircHIPK3 is significantly upregulated in CRC tissues and cell lines and is associated with the T status of tumors, lymph node and distant metastasis, and advanced clinical stage (97). High expression of circHIPK3 in CRC is an independent prognostic factor for poor overall survival (OS). Compared with paired noncancerous tissues, GC tissues exhibit significant downregulation of hsa_circ_0000096; moreover, the AUC increased from 0.82 to 0.91 when hsa_circ_0000096 was combined with hsa_circ_002509 (148). Analysis of 90 paired samples of NSCLC and adjacent normal tissues showed that hsa_circ_0014130 was significantly upregulated in NSCLC and was found to be related to tumor size, TNM stage, and lymphatic metastasis (158). ROC analysis indicated that hsa_circ_0014130 might be a prognostic biomarker in NSCLC.

In addition to identifying the differential expression of hsa_circ_002059 in GC and adjacent tissues, Li et al. (159) also found that the expression levels of hsa_circ_002059 in 36 pairs of matched plasma samples from preoperative and postoperative GC patients were significantly different. Considering that circRNAs can be secreted into the tumor microenvironment and the circulatory system by tumor cells, it is reasonable to speculate that circRNAs can be used as biomarkers to evaluate the tumor-bearing status of patients. As mentioned above, circHIPK3 can regulate the autophagy process in lung cancer through the miR124-3p–STAT3–PRKAA/AMPKa axis (85). In addition, researchers found that the ratio (C:L ratio) of circHIPK3 to linear HIPK3 (linHIPK3) can reflect the level of autophagy in cells—a higher C:L ratio indicates a lower level of autophagy and poorer prognosis.

### circRNAs Predict Not Only Tumor Metastasis but Also Drug Resistance

Recent studies have also reported that circRNAs can predict tumor metastasis and drug resistance. Xu et al. (160) analyzed the circRNA expression profile of three pairs of CRC patients with or without liver metastasis. Further verification demonstrated that circRNA_0001178 and circRNA_0000826 were significantly upregulated in metastatic CRC (CRC-m) tissues. The AUCs were 0.945 for circRNA_0001178 and 0.816 for circRNA_0000826, and both two circRNAs could differentiate liver metastases from CRC. CircASAP1 is highly expressed in the tissues of HCC patients with pulmonary metastases after curative resection (77), and its expression is positively correlated with the levels of CSF-1, MAPK1, and CD68^+^ tumor-associated macrophage. Patients with high circASAP1 expression tend to have relatively low OS and high recurrence rates. Therefore, circASAP1 can be used as a prognostic indicator for HCC.

In addition, cisplatin is one of the main chemotherapeutic drugs used to treat GC. Huang et al. (161) analyzed the correlation between the clinical efficacy of cisplatin and circRNA expression in 105 cisplatin-treated patients, and they found that the expression of circAKT3 in cisplatin-resistant patients was higher than that in cisplatin-sensitive patients. CircAKT3 is an effective predictive biomarker for cisplatin resistance in GC patients (AUC = 0.91). Confirmation of the relationships between circRNA expression and drug sensitivity would provide guidance for reasonable clinical medication and thus improve patient prognosis. Additional studies are listed in Table 4.

**Table 4:** CircRNAs as prognostic biomarkers in cancer (cohort size ≥50).

**Cancer type**	**CircRNA**	**Source**	**Cohort size**	**Expression in cancer**	**Clinical significance**	**References**
Hepatocellular carcinoma	circADAMTS13	Tissue	102	Down	Negatively associated with tumor size, BCLC stage; Positively associated with RFS	(156)
	SCD-circRNA 2	Tissue	151	Up	Negatively associated with RFS and OS; Positively associated with RFS	(162)
	circRNA_100338	Tissue	80	Up	Negatively associated with OS; Positively associated with TNM stage, vascular invasion and lung metastasis	(163)
	circRNA 101368	Tissue	51	Up	Negatively associated with OS; Positively associated with distant metastasis, TNM stage, tumor size	(164)
	circ-10720	Tissue	381	Up	Negatively associated with survival; Positively associated with clinical stage, pathology grade, AFP level and hepatitis B markers	(165)
	circSMARCA5	Tissue	197	Down	Negatively associated with TNM stage, tumor invasion and tumor size; Positively associated with tumor differentiation	(145)
Gastric cancer	circLARP4	Tissue	80	Down	Negatively associated with tumor size and lymphatic metastasis; Positively associated with OS	(104)
	hsa_circ_0001368	Tissue	68	Down	Positively associated with prognosis	(166)
	circPVT1	Tissue	187	Up	Positively associated with OS and DFS	(28)
	circNRIP1	Tissue	80	Up	Negatively associated with OS and DFS; Positively associated with tumor size and lymphatic invasion	(76)
	circ-KIAA1244	Plasma	87	Down	Negatively associated with OS; Positively associate with TNM stage and lymphatic metastasis, and poor overall survival time	(167)
	circAKT3	Tissue	149	Up	Positively associate with cisplatin resistance	(161)
Lung cancer	ciRS-7	Tissue	132	Up	Negatively associated with OS and DFS; Positively associated with tumor size, lymph node metastasis and TNM stage	(168)
	circ-PRMT5	Tissue	90	Up	Negatively associated with OS and progression-free survival; Positively associated with tumor size, lymph node metastasis and TNM stage	(169)
	circPTK2	Tissue	73	Down	Positively associated with metastatic NSCLC Tissues	(93)
	circ_0016760	Tissue	83	Up	Negatively associated with OS; Positively associated with TNM stage and lymph node metastasis	(170)
	hsa_circ_0000064	Tissue	50	Up	Positively associated with TNM stage and lymphatic metastasis	(171)
Breast cancer	hsa_circRNA_002178	Tissue	70	Up	Negatively associated with survival	(172)
	circ_0005230	Tissue	76	Up	Negatively associated with OS; Positively associated with tumor size, TNM stage and lymph node metastasis	(173)
	circKIF4A	Tissue	240	Up	Negatively associated with survival	(108)
	circGFRA1	Tissue	51	Up	Negatively associated with survival	(157)
Colorectal cancer	CircCCDC66	Tissue	229	Up	Negatively associated with OS	(96)
	circHIPK3	Tissue	178	Up	Negatively associated with OS; Positively associated with metastasis and advanced clinical stage	(97)
	circVAPA	Tissue	60	Up	Positively associated with lympho-vascular invasion, depth of invasion, lymph node metastasis, distant metastasis and TNM stage	(152)
Bladder cancer	circMTO1	Tissue	117	Up	Negatively associated with OS and DFS; Positively associated with lymph node metastasis	(174)
	cTFRC	Tissue	220	Up	Negatively associated with prognosis; Positively associated with tumor grade and T stage	(175)
Pancreatic cancer	circ-IARS	Tissue	79	Up	Negatively associated with survival; Positively associated with liver metastasis, vascular invasion, and TNM stage	(176)
	circ-PDE8A	Plasma exosomes	56	Up	Negatively associated with survival; Positively associated with duodenal invasion, vascular invasion, T factor and TNM stage	(139)
Ovarian cancer	circPLEKHM3	Tissue	86	Down	Positively associated with OS and RFS	(118)
	circWHSC1	Tissue	92	Up	Negatively associated with differentiation	(121)
Osteosarcoma	hsa_circ_0081001	Tissue	82	Up	Negatively associated with clinical outcome	(177)
Nasopharyngeal carcinoma	CDR1as	Biopsy	64	Up	Positively associated with survival	(133)
Glioma	circ_0034642	Tissue	68	Up	Negatively associated with survival; Positively associated with tumor size and advanced WHO grade	(178)

*AFP, alpha-fetoprotein; BCLC, Barcelona Clinic Liver Cancer; circRNA, circular RNA; DFS, disease-free survival; OS, overall survival; RFS, recurrence-free survival*.

## Conclusions and Perspectives

As the understanding of circRNAs has increased, their perception by the scientific community has changed dramatically. CircRNAs are not “splicing noise” but rather a class of structurally stable RNA molecules with multiple biological functions. circRNAs are generally derived from back-splicing of pre-mRNA and are widely expressed across biological systems.

The relationship between circRNAs and cancer has recently become an area of research interest. Numerous circRNAs are dysregulated and play regulatory roles in the development of cancer. Several examples of research on the roles of circRNAs in cancer are given above. In summary, circRNAs, through various signaling pathways, can participate in and affect processes related to cell proliferation, migration and invasion, apoptosis, autophagy, and drug resistance, as well as others. This capacity has inspired researchers to consider the therapeutic possibilities of targeting circRNAs and their associated pathways. In addition, researchers have also tried to synthesize artificial circRNAs for disease treatment. For example, an artificial circRNA with eight miRNA-122 binding sites was used to competitively bind to miRNA-122, which is required for the hepatitis C virus (HCV) life cycle, thereby inhibiting the propagation of HCV, with an efficiency comparable to that of the anti-miR drug miravirsen (179). This study proposed the idea that engineered circRNAs could be used for disease-specific treatments, similar to targeted drugs; however, this idea is far from clinical translation. Due to their stable structure, conservative sequence, and specific expression pattern, circRNAs have the potential to be used as biomarkers for cancer. circRNAs have wide clinical application prospects—from diagnostic assessments to predicting patient prognosis and treatment response. However, most related studies have been single-center and retrospective works.

The study of circRNAs is just beginning, and the mechanism of circRNA biogenesis is not well understood. Even though a few circRNAs have been functionally characterized, our understanding of circRNAs remains incomplete. In addition to the identified functions as miRNA sponges, protein scaffolds, translation templates, and transcriptional regulators, other mechanisms are awaiting discovery, and the cooperation and the relative importance of these mechanisms cannot be evaluated at present. In addition, the transport and degradation mechanisms of circRNAs are poorly understood. Therefore, much work remains to be done. The discovery of circRNAs has undoubtedly enriched the content of RNA regulatory networks and has offered new approaches for the development of clinically translatable diagnostic/prognostic biomarkers and therapeutic targets for cancer.

## Author Contributions

JL, XZ, and MY collected literatures and wrote the manuscript. HL contributed to writing design and revised the manuscript. All authors read and approved the final manuscript.

## Conflict of Interest

The authors declare that the research was conducted in the absence of any commercial or financial relationships that could be construed as a potential conflict of interest.
